# Monitoring the Response of Hyperbilirubinemia in the Mouse Brain by In Vivo Bioluminescence Imaging

**DOI:** 10.3390/ijms18010050

**Published:** 2016-12-28

**Authors:** Isabella Manni, Giuliana Di Rocco, Salvatore Fusco, Lucia Leone, Saviana Antonella Barbati, Carmine Maria Carapella, Claudio Grassi, Giulia Piaggio, Gabriele Toietta

**Affiliations:** 1 Department of Research, Advanced Diagnostic, and Technological Innovation, Regina Elena National Cancer Institute, 00144 Rome, Italy; isabella.manni@ifo.gov.it (I.M.); giuliana.dirocco@ifo.gov.it (G.D.R.); giulia.piaggio@ifo.gov.it (G.P.); 2 Institute of Human Physiology, Medical School, Università Cattolica del Sacro Cuore, 00168 Rome, Italy; salvatore.fusco@unicatt.it (S.F.); lucia.leone@unicatt.it (L.L.); saviana.barbati@rm.unicatt.it (S.A.B.); claudio.grassi@unicatt.it (C.G.); 3 Department of Neurosurgery, Regina Elena National Cancer Institute, 00144 Rome, Italy; carmine.carapella@ifo.gov.it

**Keywords:** bevacizumab, bilirubin, bilirubin-induced neurologic dysfunction, blood–brain barrier, in vivo bioluminescence imaging, hyperbilirubinemia, kernicterus, luciferase, transgenic mice

## Abstract

Increased levels of unconjugated bilirubin are neurotoxic, but the mechanism leading to neurological damage has not been completely elucidated. Innovative strategies of investigation are needed to more precisely define this pathological process. By longitudinal in vivo bioluminescence imaging, we noninvasively visualized the brain response to hyperbilirubinemia in the MITO-Luc mouse, in which light emission is restricted to the regions of active cell proliferation. We assessed that acute hyperbilirubinemia promotes bioluminescence in the brain region, indicating an increment in the cell proliferation rate. Immunohistochemical detection in brain sections of cells positive for both luciferase and the microglial marker allograft inflammatory factor 1 suggests proliferation of microglial cells. In addition, we demonstrated that brain induction of bioluminescence was altered by pharmacological displacement of bilirubin from its albumin binding sites and by modulation of the blood–brain barrier permeability, all pivotal factors in the development of bilirubin-induced neurologic dysfunction. We also determined that treatment with minocycline, an antibiotic with anti-inflammatory and neuroprotective properties, or administration of bevacizumab, an anti-vascular endothelial growth factor antibody, blunts bilirubin-induced bioluminescence. Overall the study supports the use of the MITO-Luc mouse as a valuable tool for the rapid response monitoring of drugs aiming at preventing acute bilirubin-induced neurological dysfunction.

## 1. Introduction

Severe hyperbilirubinemia, due to high levels of unconjugated bilirubin, may cause bilirubin encephalopathy, also known as kernicterus. This condition is characterized by irreversible and selective brain damage or by a broad spectrum of less severe and defined neurologic alterations, referred to as bilirubin-induced neurological dysfunction (BIND) [1,2]. In the blood, more than 99% of unconjugated bilirubin (UCB) is bound to albumin and only a small fraction (less than 1%) remains unbound (defined as UCB_FREE_). Only UCB_FREE_, and not albumin-bound UCB, is able to cross the blood–brain barrier (BBB), and it is, therefore, responsible for bilirubin-induced neurotoxicity. Factors which increase UCB_FREE_, such as hypoalbuminemia, hyperbilirubinemia, and the presence of drugs which displace bilirubin from its binding sites on albumin, as well as BBB damage, have a clinical relevance in bilirubin-induced neurotoxicity. Recent evidence suggests that several transporters present on the BBB may play an active role in limiting UCB_FREE_ diffusion into the brain [3]. In addition, severe hyperbilirubinemia may induce BBB endothelial disruption [4], possibly exposing the neurons to both UCB_FREE_ and albumin-bound UCB [5].

How UCB exerts its neurotoxic effect is not fully clear [6]. Nonetheless understanding the cellular events leading to bilirubin-induced neurological dysfunction is essential to the development of strategies aimed at preventing the onset of kernicterus. In vitro studies have demonstrated that bilirubin induces plasma membrane and mitochondrial damage in neural and glial cells [7,8,9]. Proteomic analysis on neuronal cells exposed to UCB revealed altered expression of proteins involved in cell proliferation, protein degradation, and oxidative stress response [10]. Moreover UCB triggers the inflammatory signaling pathway in astrocytes through the release of pro-inflammatory cytokines [11] and activates phagocytic and inflammatory phenotypes in microglial cells [12]. Although valuable information has been collected, in vitro studies are largely limited by the experimental conditions, including cell isolation procedures, method of cell culture, and dose response to bilirubin supplementation in the culture medium. Therefore in vivo studies on suitable animal models are needed to more precisely define the physiological and pathological processes related to hyperbilirubinemia. In vivo studies to determine bilirubin toxicity rely predominantly on the hyperbilirubinemic Gunn rat, an animal model for Crigler–Najjar syndrome, characterized by an inherited deficiency of hepatic bilirubin glucuronidation. In the Gunn rat, induction of hemolysis by phenylhydrazine administration results in increased levels of UCB, leading to neurological abnormalities similar to kernicterus [13]. The same treatment induces brain damage also in wild type Wistar rats [14]. Recently combined administration of phenylhydrazine and sulfisoxazole to produce hypo-albuminemia was described for the induction of kernicterus in Wistar rats [15].

In the current work, we explored in vivo the effects of experimental hyperbilirubinemia in the MITO-Luc mouse [16]. In this animal model, luciferase activity is restricted to proliferating cells being under the control of the nuclear factor-Y (NF-Y) transcription factor. Therefore, in this transgenic mouse, bioluminescence imaging (BLI) can be used as simple and sensitive surrogate marker for the identification of the regions of active cell proliferation. In MITO-Luc mice in particular, high bioluminescence can be detected in tissues proliferating under normal physiological conditions such as spleen, testis, and bone marrow (vertebral column, sternum, and femur), while non-proliferating tissues such as lung, brain, heart, aorta, skeletal muscle, liver, and kidney do not emit light [16]. In addition, we and others have also demonstrated that, in the MITO-Luc mouse, bioluminescence correlates with cell proliferation subsequent to drug-mediated tissue toxicity [16,17] or induced by ischemic stroke injury [18].

The purpose of the study is to support the use of the MITO-Luc mouse as a model for understanding the pathophysiological mechanism of hyperbilirubinemia and for providing a valuable pharmaceutical tool for screening drug development and therapy response monitoring for bilirubin-induced neurotoxicity.

## 2. Results

### 2.1. Hyperbilirubinemia Induces Bioluminescence in the Brain of MITO-Luc Mice

**Figure 1:**
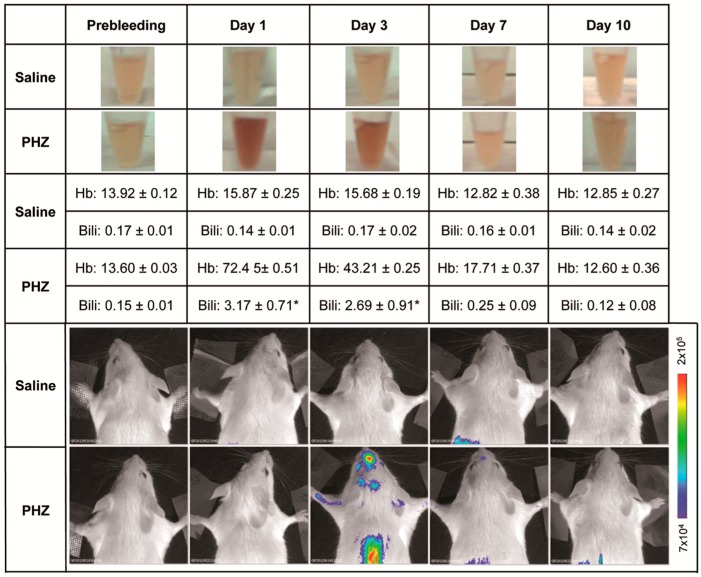
Effect of phenylhydrazine administration into MITO-Luc mice. Saline solution (saline) as control or phenylhydrazine (PHZ) (75 mg/kg) was administered via intra peritoneal route to MITO-Luc mice (*n* = 5 per group) to induce experimental hemolysis. At different time points, we collected blood samples by retro-orbital bleeding. The figure shows photographs of about 20 µL of serum from a representative animal from each group (top rows) and serum levels of hemoglobin (Hb, expressed in g/dL) and total bilirubin (Bili, expressed in mg/dL) (middle rows). Blood samples collected from the animals 3 days before treatment (referred as “prebleeding” in figure) were used as physiological baseline control. Data are mean ± SEM. Normal clinical chemistry values are: total bilirubin 0.1–0.7 mg/dL; hemoglobin 12–16 g/dL. Due to the fact that hemolysis interferes with accurate bilirubin determination, values indicated with an asterisk (*) should be considered approximated values. The bottom part of the figure shows the in vivo bioluminescence imaging of a representative animal for each group performed at the same time points. The color bar and numbers next to the image illustrate the relative bioluminescent signal intensities from the lowest (blue) to the highest (red), with minimal and maximal values expressed in photons per second per square centimeter per steradian (photons/s/cm^2^/sr).

Bilirubin is a product of heme catabolism. Phenylhydrazine (PHZ) is a strong oxidant agent and a potent hemolytic known to cause anemia [19]. Phenylhydrazine-mediated hemolysis induces hepatic heme oxygenase, which, in turn, causes hyperbilirubinemia. A group of 5 MITO-Luc mice were administered, by intra peritoneal injection, with PHZ, 75 mg/kg/day for 2 consecutive days, as previously described for the induction of experimental hemolysis to study bilirubin encephalopathy in the rat brain [14]. Marked transient hemolysis was observed in MITO-Luc mice beginning at day 1, after the last PHZ injection, and resolved to normal values in approximately 1 week (Figure 1). Hemolysis interferes with accurate determination of bilirubin; nonetheless increased levels of bilirubin were also observed, with a kinetic similar to that of the serum hemoglobin (Figure 1). No significant alteration of hemoglobin and bilirubin values was observed in the control group (*n* = 5), which was receiving saline solution by the same route of administration (Figure 1).

**Figure 2:**
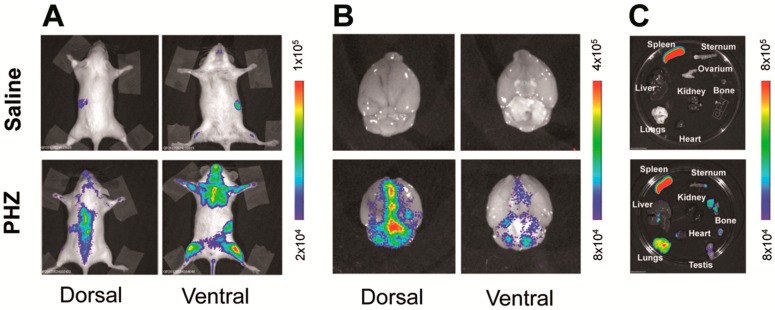
Phenylhydrazine administration modulates bioluminescence in vivo and ex vivo in MITO-Luc mice. MITO-Luc mice receiving intra peritoneal administration of saline solution (saline) or phenylhydrazine (PHZ) 75 mg/kg for two consecutive days were analyzed by in vivo and ex vivo bioluminescence imaging (BLI) 3 days after the last PHZ administration. In particular, the figure shows in vivo BLI analysis of a representative animal from the control (top) and PHZ (bottom) treated groups (*n* = 3) (**A**); ex vivo BLI analysis of brains (**B**) and other organs (**C**) dissected from the same animals after necropsy. The color bars represent bioluminescent signals in radiance (photons/s/cm^2^/sr) from the lowest (blue) to the highest (red).

Interestingly increased levels of hemoglobin and bilirubin in the serum of the PHZ treated MITO-Luc mice were associated with increased light emission, which reached the maximum intensity 3 days after the end of the treatment (Figure 1). In MITO-Luc mice, only organs in active proliferation such as bone marrow, testis, and spleen are positive by BLI analysis. Luciferase activity is also detected in regions undergoing continuous damage and regeneration such as teeth and paws [16]. Quiescent organs such as liver, brain, heart, aorta, and lungs do not emit light. Accordingly, in the current study, we observed that the BLI signal in the area of the brain was negligible and comparable to background levels in the saline-treated control mice, while a signal was determined in all animals administered with PHZ (Figure 2A,B). Although light emission in the brain was rather diffuse and difficult to precisely localize with the present method of analysis, the highest BLI signals in PHZ-treated mice were located in the longitudinal fissure that separates the two cerebral hemispheres and at the convergence with the transversal fissure that separates the hemispheres from cerebellum. However we cannot exclude that localization on the originating signals can be confined to the intravascular or perivascular space (Figure 2B).

PHZ targets the hematopoietic system [20], inducing anemia and activation of the immune system, triggering phagocytosis in spleen and liver [21]. In addition, PHZ-associated generation of reactive oxidative species causes vascular dysfunction in the lungs [22]. Indeed lungs and livers were negative by in vivo and ex vivo BLI analysis in MITO-Luc mice treated with saline solution (total flux below 1.5 × 10^5^ photons/s/cm^2^/sr), while a detectable signal (total flux 8.1 × 10^6^ ± 3.8 × 10^5^ photons/s/cm^2^/sr) was noticeable in the lungs and, to a lesser extent (total flux 1.9 × 10^6^ ± 6.7 × 10^4^ photons/s/cm^2^/sr), in the livers of PHZ-treated animals (Figure 2A,C). This result, therefore, indirectly confirms that PHZ-induced hemolytic anemia is associated with vascular damage to lungs and liver [22].

**Figure 3:**
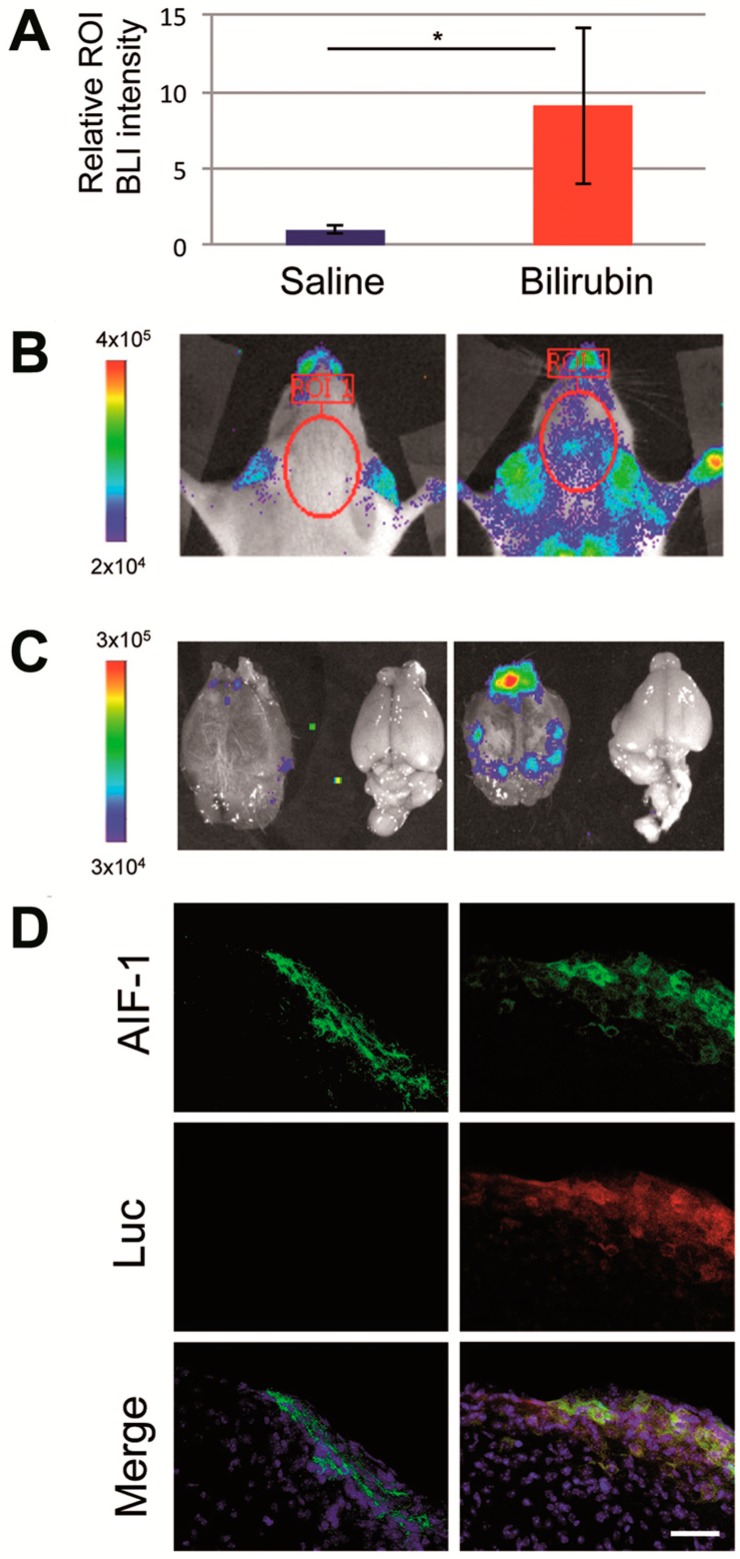
Effects of intra peritoneal injection of bilirubin into MITO-Luc mice. Representative MITO-Luc mouse (*n* = 5 per group) receiving saline solution or bilirubin (50 mg/kg) were analyzed 1 day after administration. (**A**) Relative bioluminescence intensity assessed by Living Imaging software; the asterisk (*) indicates a significant difference versus the control group (*p* ≤ 0.05); (**B**) bioluminescence imaging (BLI) of live animals; (**C**) BLI imaging of brain after necropsy. The color scale next to the images indicates radiance, with red and blue respectively representing the highest and lowest bioluminescent signals, expressed in photons/s/cm^2^/sr; (**D**) Representative immunohistochemical images of lateral sagittal brain sections including middbrain and substantia nigra from saline- (**left** panels) and bilirubin-treated mice (**right** panels), stained with antibodies anti-allograft inflammatory factor 1 (AIF-1, green), antibodies anti-luciferase (Luc, red), and relative merged images with 4′,6-diamidino-2-phenylindole (DAPI, blue) staining. Scale bar: 100 µm.

As an alternative way to PHZ administration to induce hyperbilirubinemia in the MITO-Luc mice, we injected a solution of commercially available purified bilirubin. Longitudinal BLI analysis was performed as described above. In the group of animals treated with bilirubin we detected an increment of the bioluminescence compared with the saline-treated animals used as control (Figure 3A). As in the PHZ treated mice, we observed the presence of luciferase signal assessed by BLI analysis in correspondence with the brain area (Figure 3B). In particular, we determined the light emission in the brain area in mice before the treatment and considered this value as background level. Then we determined BLI in the same area 1 day after saline solution or bilirubin administration and subtracted the background level. The value of BLI emission from the brain region in bilirubin treated mice was higher than in PBS treated mice (*p* = 0.02) (Figure 3A,B). This increment in bioluminescence suggests higher cell proliferation, which is correlated to the toxic effect associated with the raise of bilirubin to supraphysiological levels.

BLI was also performed after necropsy on brains of selected animals sacrificed 1 day after administration (Figure 3C), confirming a stronger signal in bilirubin-injected animals. Immunohistochemical analysis of the sagittal brain sections of these mice revealed the presence of luciferase positive cells in bilirubin-treated mice, not detectable in control animals (Figure 3D). Microglia activation has been observed in the Gunn rat, the animal model of Crigler–Najjar syndrome, a genetic disorder characterized by unconjugated hyperbilirubinemia [23]. Accordingly it has been proposed that an acute neuronal injury, such as the one associated with hyperbilirubinemia, might promote microglia activation and transient massive proliferation of the resident microglial population and the recruitment of a smaller subpopulation of bone marrow-derived microglia [24]. In order to identify the cell type responsible for BLI emission upon hyperbilirubinemia, we performed double-staining immunohistochemistry using antibodies anti-luciferase and anti-allograft inflammatory factor 1 (AIF-1, also referred as ionized calcium-binding adapter molecule 1, Iba1) on brain sections of control and bilirubin-treated mice (Figure 3D). AIF-1/Iba1 is a marker for microglial cells, which are considered the resident macrophages of the brain. AIF-1 plays a role in the activation and function of microglia and its expression is up-regulated in response to vascular injury and neuroinflammation [25]. Therefore the increased presence of luciferase and AIF-1 double positive cells identified in the sections of brains isolated from hyperbilirubinemic animals suggests that microglial cell proliferation is, at least in part, responsible for the increased luciferase activity observed by in vivo imaging.

### 2.2. Modulation of Bilirubin/Albumin Binding Impinges on Bilirubin-Induced Bioluminescence in MITO-Luc Mice

Albumin-bound bilirubin cannot translocate across the BBB. Accordingly albumin infusion has been proposed to reduce the risk of kernicterus in acute phases of hyperbilirubinemia [26] and in hyperbilirubinemic neonates requiring exchange transfusion, but the efficacy of the treatment is controversial [27].

We wanted to examine whether and to what extent albumin administration, possibly reducing the UCB_FREE_ levels, may modify the pattern of cell proliferation observed in MITO-Luc mice subjected to hyperbilirubinemia. Conversely we also tested the effect of the administration of sulphadimethoxine, an antibiotic known to displace bilirubin from its binding sites on albumin, consequently increasing the levels of UCB_FREE_ [28].

**Figure 4:**
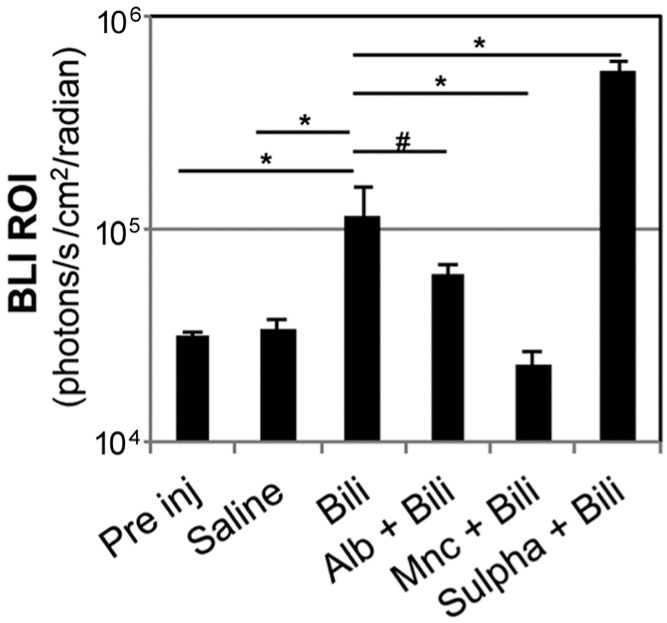
Effect of different pharmacological treatments on brain bioluminescence modulation. MITO-Luc mice received, in conjunction with bilirubin injection, one of the following treatments: daily intra peritoneal (i.p.) administration of human serum albumin (Alb) (2.5 g/kg body weight) for 2 days before bilirubin (Bili) administration (Alb + Bili group); administration of minocycline (Mnc) (50 mg/kg) i.p. 30 min before and 2 h after bilirubin injection (Mnc + Bili group); i.p. administration of sulphadimethoxine (Sulpha) (200 mg/kg body weight) 2 h before bilirubin administration (Sulpha + Bili group). Quantification of BLI signals in the regions of interest in the brain area were quantified 5 h after bilirubin administration and compared to the signal assessed in the same animals 3 days before the administration of the different substances (referred as “Pre inj” for “pre injection”), and with a group of animals receiving saline solution only (Saline). *n* = 25 at the pre injection assessment; then the animals were randomly divided in groups of 5 mice each. The significance of differences of the bilirubin group (Bili) vs. the controls and experimental groups are shown. In particular, (*) indicates *p* ≤ 0.05; (#) *p* > 0.05 between bilirubin and albumin + bilirubin groups.

Albumin infusion before bilirubin administration resulted in a trend of reduction of BLI in the brain area assessed 5 h after bilirubin administration (1.15 × 10^5^ ± 3.94 × 10^4^ photons/s/cm^2^/sr in bilirubin treated animals vs. 6.01 × 10^4^ ± 6.60 × 10^3^ photons/s/cm^2^/sr in mice receiving albumin infusions before bilirubin administration); nonetheless the difference in the intensity of the bioluminescence signal was not statistically significant in the two groups (*p* = 0.2927) (Figure 4). Therefore, in this experimental setting, albumin infusion was not sufficient to prevent increased bioluminescence associated with hyperbilirubinemia.

Conversely sulphadimethoxine administration before bilirubin administration resulted in a statistically significant increase of BLI in the brain area compared to animals not receiving the treatment before induction of hyperbilirubinemia (Figure 4). Moreover sulphadimethoxine-treated mice developed signs consistent with bilirubin encephalopathy such dystonia and lethargy. For this reason, they were euthanized the day after administration. These data are in agreement with the ability of sulphadimethoxine to displace bilirubin from its binding sites on albumin, therefore decreasing the levels of albumin-bound UCB leading to exacerbation of bilirubin acute toxicity [29].

### 2.3. Minocycline Treatment Blunts Hyperbilirubinemia-Induced Bioluminescence in MITO-Luc Mice

Minocycline is a synthetic tetracycline which has been shown to be neuroprotective against bilirubin-induced brainstem auditory evoked potentials in the Gunn rat [28]. The potential neuroprotective role of minocycline has been ascribed to its ability to reach the brain parenchyma due to its high lipid solubility and to inhibit microglial activation and migration of macrophages from the periphery to the central nervous system [30,31]. Microglia cells are the resident innate immune cells on the central nervous system. In vivo studies suggest that modulation of microglial activation might be a promising target against bilirubin encephalopathy [12]. Activation of microglia cells occurs within a few hours after a neurologic insult, leading to the up-regulation of several inflammatory mediators and to BBB damage. Therefore minocycline-induced inhibition of microglia activation leads to anti-inflammatory anti-apoptotic effects in addition to an anti-oxidant effects [32], which alone are not sufficient to provide neuroprotection [33]. We tested the consequences of the administrations of minocycline 1 h before and 2 h after treatment with bilirubin to induce hyperbilirubinemia in the MITO-Luc mouse model. In particular, we determined by BLI that induction of bioluminescence in the brain area observed several hours after bilirubin administration was blunted by minocycline treatment (Figure 4). This result is in line with the beneficial effect of minocycline treatment in several animal models of central nervous system disorders including cerebral ischemia and Parkinson’s disease [30].

### 2.4. Modulation of Blood–Brain Barrier Permeability Affects Bioluminescence in the Brain Region in Hyperbilirubinemic MITO-Luc Mice

As mentioned above, unconjugated bilirubin (UCB) can enter the brain if it is not bound to albumin in the blood (UCB_FREE_) or if the blood–brain barrier (BBB) has been damaged. We wanted to examine whether and to what extent the alteration of BBB permeability has an effect on the modulation of the luciferase activity in the brain region observed in the MITO-Luc mice after induction of hyperbilirubinemia.

**Figure 5:**
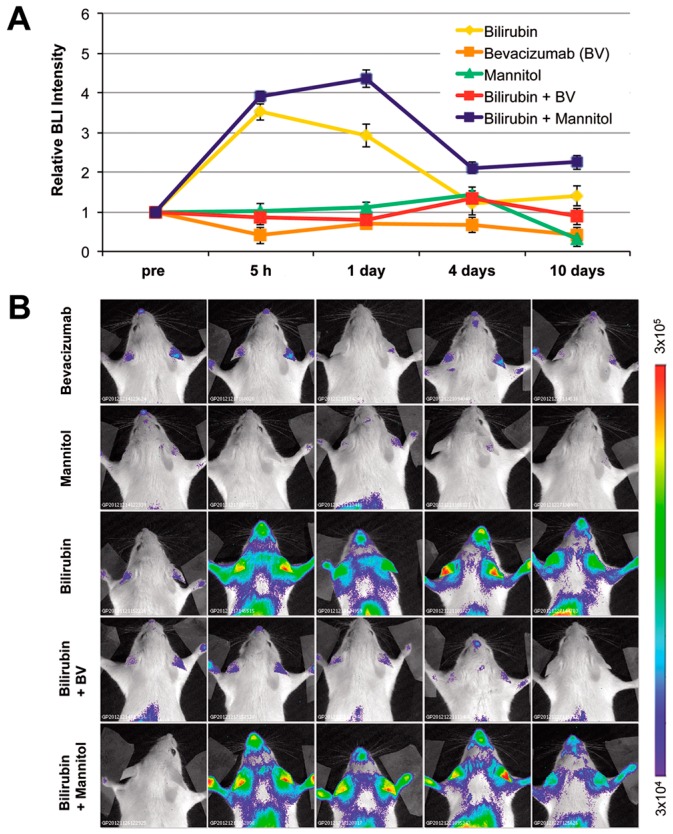
Effect of alteration of blood–brain barrier permeability. Before bilirubin administration, MITO-Luc mice were pretreated with mannitol (3 mL of 25% mannitol/100 g body weight) or bevacizumab (25 mg/kg body weight) and bioluminescence imaging analysis (BLI) was performed at different time points. (**A**) Quantification of BLI signals in selected regions of interest in the brain area. Statistical analysis revealed significant differences (*p* ≤ 0.05) of the bilirubin group vs. bilirubin + bevacizumab group at the 5 h and 1 day time points; (**B**) BLI analysis of one representative animal out of five per group, at the different time points. The color bar and numbers illustrate the relative bioluminescent signal intensities from the lowest (blue) to the highest (red), with minimal and maximal values expressed in photons/s/cm^2^/sr.

Systemic administration of mannitol has been used to reversibly perturb the BBB permeability to improve delivery into the brain of chemotherapeutic drugs, gene therapy vectors, and neuronal stem cells [34,35]. Right after a bolus administration of mannitol, circulating volume raises, blood viscosity decreases, and, therefore, cerebral blood flow increases. In addition, mannitol administration results in alterations of brain osmolarity, with reduction of intracranial pressure leading to improved blood–brain barrier permeability. Therefore mannitol administration is associated with an increase in BBB permeability to intravascular substances. We administered mannitol to a group of MITO-Luc mice before bilirubin injection. This treatment resulted in an increment of the BLI signal in the brain area determined 5 h after bilirubin administration, compared with animals not receiving mannitol injection (Figure 5). On the other hand, in mice receiving only saline solution and in mice receiving only mannitol, we were not able to detect any difference in the BLI profile compared to baseline levels. Therefore mannitol-associated BBB disruption, leading to an improved delivery of bilirubin into the brain, augmented the BLI signal observed in hyperbilirubinemic MITO-Luc mice.

Conversely bevacizumab (ATC code: L01XC07), a recombinant monoclonal antibody with high affinity to the human vascular endothelial growth factor (VEGF) used for angiogenesis inhibition, for the treatment of several forms of cancers, induces vascular stabilization and reduces BBB permeability [36]. We administered bevacizumab (Avastin^®^) to MITO-Luc mice 1 h before bilirubin injection. Mice treated with bevacizumab before bilirubin administration did not show any significant increment in BLI levels (Figure 5). Therefore bevacizumab-associated BBB stabilization, leading to reduced BBB permeability to bilirubin, resulted in no significant alteration of the level of proliferation in hyperbilirubinemic MITO-Luc mice.

Taken together, these data suggest that induction of hyperbilirubinemia in the MITO-Luc mouse results in an increase in the proliferative status in the brain region, which can be easily and noninvasively detected by BLI imaging. The appearance of this bioluminescence signal correlated with the possibility of UCB_FREE_ crossing the BBB and activating the microglia.

## 3. Discussion

In neonatal jaundice and in Crigler–Najjar syndrome, a hereditary error of bilirubin metabolism, high blood levels of unconjugated bilirubin (UCB) may induce neurological dysfunction (BIND) associated with minor brain deficit or with severe life-threatening bilirubin encephalopathy (kernicterus) [2]. The mechanisms by which UCB exerts its harmful effect on the brain are complex and not fully understood [37]. Several factors are determinant in patients’ susceptibility to bilirubin-induced neuronal damage including the levels of circulating UCB, the binding of bilirubin to albumin, and, consequently, the levels of the unbound fraction of unconjugated bilirubin. A key role in the dynamic exchange between blood and brain is played by the blood–brain barrier (BBB); therefore BBB permeability and its functional maturity are very important in neuroprotection [6]. Moreover inflammation is one of the leading causes in the disturbance of BBB integrity [38].

Studies performed on isolated cells have provided evidence that possible targets of UBC toxicity include neurons, glial cells, astrocytes, brain microvascular cells, and erythrocytes [4,25,39,40]. At the molecular level, high bilirubin concentration directly causes alteration in mitochondrial, plasma, and endoplasmic reticulum membranes [41]. Perturbations of membrane permeability result in mitochondrial dysfunction, increased intracellular calcium concentration, alteration of glucose metabolism, loss of DNA, and protein synthesis. These events ultimately lead to cell death by either apoptosis or necrosis. Innovative models are required to further clarify the pathogenesis of neuronal damage in response to hyperbilirubinemia. In particular, it would be critical to determine the specific temporal progression in vivo of acute bilirubin-induced neurotoxicity.

Bioluminescence imaging involves the detection of photons from cells expressing a luciferase enzyme in the presence of a suitable luciferin substrate. This procedure offers various advantages over alternative molecular imaging techniques, including a very high signal to background ratio allowing for more sensitive and quantitative analysis. The possibility to noninvasively perform BLI allows for repetitive analysis on the same individual, making longitudinal studies possible. We have recently developed a mouse model (named MITO-Luc) in which luciferase expression is driven by the cyclin B2 promoter and therefore is restricted to proliferating cells. In physiological condition, the MITO-Luc mouse brain does not emit a detectable bioluminescence signal [16]. The blood–brain barrier reduces diffusion of the luciferase substrate [42]. Nonetheless BBB osmotic disruption by mannitol administration does not alter BLI signal in MITO-Luc mice (Figure 5); therefore the lack of bioluminescence signal in normo-bilirubinemic animals is due to the absence of luciferase-expressing cells in the quiescent brain and is not merely due to poor d-luciferin permeability to BBB.

In the present manuscript, taking advantage of the MITO-Luc mouse model, through in vivo imaging we provide indirect evidence that acute hyperbilirubinemia results in the appearance of proliferating cells in the brain region. We were able to determine the kinetics of cell proliferation in response to hyperbilirubinemia in a real-time manner, providing a unique tool for rapid response monitoring of drugs aimed at preventing acute bilirubin-induced neurological dysfunction. In fact, activation of NF-Y driven luciferase gene activity in the brain area of the MITO-Luc mouse model that has undergone induction of hyperbilirubinemia may, in our opinion, represent a simply assessable and sensitive tool for dissecting the pathophysiological events linking hyperbilirubinemia to neuronal damage. Interestingly phenomena such as pharmacological displacement of bilirubin from its albumin binding sites, administration of drugs known to inhibit microglial activation/proliferation, and modulation of the blood–brain barrier permeability, known to impact on bilirubin induced neurological damage onset, have a dramatic effect on the development of bioluminescence signal in our experimental model. In particular, albumin infusion has been proposed to reduce the risk of kernicterus in acute phases of hyperbilirubinemia in newborns, but there is a lack of general consensus on the efficacy of this treatment [27]. Controversial results have also been obtained in in vitro toxicological studies, due, at least in part, to the fact that bilirubin-albumin binding may be affected by the source of albumin used to perform the assay, since adult albumin has an increased bilirubin-binding capacity compared to that obtained from newborns [43]. In our experimental model, we observed a trend of reduction of the induction of NF-Y-driven luciferase bioluminescence in the group of animals treated with albumin infusion, but the difference was not statistically significant. This may suggest that albumin infusion alone may not be sufficient to prevent bilirubin-induced damage. On the other hand, since our study was performed on adult mice, this model may not be fully representative of neonatal hyperbilirubinemia characterized by reduced expression of bilirubin efflux transporters such as P-glycoprotein [3].

We explored alternative pharmacological strategies aimed at preventing bioluminescence associated with cell proliferation subsequent to acute hyperbilirubinemia. In particular, minocycline treatment has been associated with prevention of severe BBB damage, via blocking microglia activation, which, in turn, is associated with microglial cell proliferation, morphological change, and production of pro-inflammatory cytokines [32,38]. Consistently minocycline treatment is able to reduce to basal levels bioluminescence associated with bilirubin administration in the MITO-Luc mouse, suggesting a possible role of NF-Y in promoting microglial cell proliferation. As a matter of fact, NF-Y has been shown to regulate the expression of genes implicated in the maturation of macrophages [44]. In addition, in brain sections of animals administered with bilirubin, we detected, by immunohistochemistry, staining cells positive for both luciferase and the microglial marker AIF-1 (Iba1). This finding supports the hypothesis that, in our model, hyperbilirubinemia is associated with increased proliferation of microglial cells, even if we cannot exclude involvement of other brain cells including astrocytes, vascular smooth muscle cells, oligodendrocytes, and neuronal precursors. Indeed increasing evidence indicates that microglial cells proliferate in response to alterations of the central nervous system homeostasis such as hyperbilirubinemia [39], infection, and in acute and chronic neurodegenerative diseases [45]. In particular, proliferation of microglial cells within 72 h after cerebral ischemia plays an important neuroprotective role [46]. Interestingly pharmacological modulation of microglial cell proliferation has been recently been proposed as an innovative therapeutic strategy for Alzheimer’s disease [47].

Bevacizumab (Avastin) is a recombinant, anti-human vascular endothelial growth factor (VEGF), monoclonal antibody currently used in molecular-targeted therapies for some types of cancers [48]. Recently repositioning of bevacizumab has been proposed for cartilage regeneration [49]. We have observed that BBB stabilization by administration of bevacizumab [36] reduces bioluminescence output in the brain area of the MITO-Luc mouse model undergone to induce hyperbilirubinemia. This result may provide the proof of principle for a possible use of Avastin treatment for the management of subjects exposed to acute hyperbilirubinemia in order to reduce the risk of severe neurological damage, although the systemic use of bevacizumab in pediatric patients might raise safety concerns [50].

Collectively our data support the use of the MITO-Luc mouse as a tool for basic investigation aimed at elucidating the cellular and molecular mechanisms leading to neural damage associated with unconjugated hyperbilirubinemia and to translational research that can impact the development of innovative therapeutic strategies for neonatal jaundice and Crigler–Najjar syndrome. Moreover the MITO-Luc model could be instrumental for studies of other neurodegenerative pathologies such as Alzheimer’s, Parkinson’s, Huntington’s, and prion diseases, characterized by increased cellular proliferation in the central nervous system [45].

## 4. Materials and Methods

### 4.1. Experimental Animal Procedures

All experimental procedures conformed to protocols approved by the Regina Elena National Cancer Institute Animal Care and Use Committee and were performed in accordance with the Guide for the Care and Use of Laboratory Animals and the guidelines of the National Institutes of Health, according to the current National Legislation (Art. 31 D.lgs 26/2014, 4 March 2014). The animals used in the study were 6–8 weeks old MITO-Luc mice [16] of both sexes, maintained on an FVB background. The mice were housed at a constant temperature with a 12 h light/dark cycle and allowed free access to standard diet and water. Hyperbilirubinemia was achieved by induction of hemolysis by intra peritoneal (i.p.) administration of phenylhydrazine (75 mg/kg body weight) (Sigma Aldrich, St. Louis, MO, USA) for two consecutive days, as previously described [14]. Alternatively bilirubin (Sigma Aldrich) was dissolved in 0.1 N NaOH, then phosphate buffered saline pH 7.4 (PBS) was added and the solution immediately injected i.p. (50 mg/kg body weight). Additional groups received, in conjunction with bilirubin injection, one of the following treatments: daily i.p. administration of human serum albumin (Sigma Aldrich) (2.5 g/kg body weight) for 2 days before bilirubin administration (Alb + bilirubin group); i.p. administration of sulphadimethoxine (Sigma Aldrich) (200 mg/kg body weight) 2 h before bilirubin administration [28] (Sulpha + bilirubin group); i.p. administration of minocycline (Sigma Aldrich) (50 mg/kg) by i.p. administration 30 min and 2 h after bilirubin injection [28] (Mnc + Bilirubin group).

Brain osmolarity and reduction of intracranial pressure was achieved on a selected group of animals by i.p. injection of mannitol solution according to a procedure previously described [51]. In particular, a volume corresponding to 3 mL of 25% mannitol in saline solution per 100 g of body weight was administered i.p. 10 min before bilirubin administration, performed as described above. Drinking water was withheld for 5 h after mannitol administration and then water was re-administered ad libitum.

Bevacizumab (Avastin^®^, Roche, Basel, Switzerland) was administered by i.p. injection into MITO-Luc mice at 25 mg/kg 30 min before bilirubin administration. Previous pharmacological data have demonstrated that i.p. administration of this dose in mice results in plasma concentration of the drug achievable in humans after intravenous administration of a 15 mg/kg dose, the clinical dose used in patients with malignant gliomas [48].

Blood samples were collected from sedated animals via retro-orbital bleeding. Serum was obtained by centrifugation at 1500× *g* for 10 min. Direct spectrophotometry measurements of serum levels of hemoglobin and bilirubin were performed.

### 4.2. In Vivo and Ex Vivo Optical Bioluminescence Imaging

Bioluminescence imaging (BLI) analysis was performed using the IVIS Lumina II equipped with the Living Image software for data quantification (PerkinElmer, Waltham, MA, USA), as previously described [52]. For in vivo imaging in particular, mice were sedated with an i.p. injection of Avertin solution (2.2.2-tribromoethanol; Sigma Aldrich) (240 mg/kg) and d-luciferin (PerkinElmer) dissolved in PBS (75 mg/kg body weight) was administered i.p. 10 min before analysis. For ex vivo imaging, animals were euthanized and their organs excised, placed into clear bottom tissue culture dishes, and incubated 10 min before analysis into PBS containing d-luciferin (150 µg/mL) [53].

### 4.3. Immunohistological Analysis

Mice were anesthetized with a cocktail of ketamine (100 mg/mL) and medetomidine (1 mg/mL) (ratio: 5:3) and transcardially perfused with an oxygenated Ringer’s solution, pH 7.3, followed by 4% freshly depolymerized paraformaldehyde in 0.1 M PBS, pH 7.4. The brain was removed from the skull, post-fixed overnight at 4 °C, and then transferred into a solution of 30% sucrose in PBS for 2 days. Sagittal or coronal brain sections (45-μm-thick) were then cut with a vibratome (VT1000S, Leica Microsystems, GmbH, Wetzlar, Germany) and floated on ice-cold PBS. Sections were collected and stored until use in cryoprotectant at −20 °C. After three 10 min rinses in PBS, sections were incubated in a blocking solution containing 1% bovine serum albumin, 10% normal goat serum, and 0.5% Triton X-100. The sections were then incubated for 48 h at 4 °C with the primary antibody, extensively washed, and then re-incubated with the secondary antibody (AlexaFluor-546, 1:500; Life Technologies, Darmstadt, Germany), as previously described [54]. The primary antibodies used were the following: anti firefly luciferase (Sigma Aldrich), 1:200; anti Iba1 (1022-5) (Santa Cruz Biotechnology, Dallas, TX, USA), 1:200. Two-dimensional images, created as the maximum projection of z-stack images, were obtained with a Nikon A1 confocal microscope equipped with a 40× objective (Nikon Co., Ltd., Tokyo, Japan).

### 4.4. Statistical Analysis

Results are expressed as means ± standard error of the mean (SEM). Unless otherwise stated, in in vivo studies the number of mice randomly assigned in each experimental group (*n*) was at least 5. As a matter of fact, it has been recently demonstrated that, for proliferation studies using MITO-Luc mice, samples size can be reduced by virtue of the reproducible quantitative measurement of bioluminescence as a surrogate marker of cell growth [17]. Data analysis and comparisons between groups were performed with INSTAT software (GraphPad, San Diego, CA, USA). The significance of differences was assessed with a two-tailed Student *t* test for unpaired data; the statistical significance level was set at *p* ≤ 0.05. Multiple comparisons were performed using one-way analysis of variance (ANOVA), followed by Tukey’s comparison test.

## 5. Conclusions

By longitudinal BLI analysis, we determined that hyperbilirubinemia determines an increase in the bioluminescence emission in the brain region in the MITO-Luc mouse. In this model, intensification of the light intensity reflects an increase in the proliferative status. Increased bioluminescence in the brain region associated with hyperbilirubinemia was modulated by: (i) pharmacological displacement of bilirubin from its albumin binding sites; (ii) alteration of the blood brain barrier permeability; (iii) treatment with minocycline, an inhibitor of microglial activation; or (iv) with the anti-vascular endothelial growth factor antibody bevacizumab. Detection in the brain section of the cell double positive for luciferase and the microglial marker AIF1 suggests that hyperbilirubinemia is associated, at least in part, with increased proliferation of microglial cells. Based on these data, we believe that our experimental model has the potential to facilitate the determination of the kinetics of the events leading to bilirubin-induced neurologic dysfunction in a real-time manner, providing a unique tool for screening drug development, therapy response monitoring, and BBB drug delivery strategies in order to prevent BIND.

## Abbreviations

BIND	Bilirubin-induced neurological dysfunction
NF-Y	Nuclear factor-Y
Alb	Albumin
BBB	Blood–brain barrier
BLI	Bioluminescence imaging
Mnc	Minocycline
PHZ	Phenylhydrazine
UCB	Unconjugated bilirubin
